# Desmoglein 3, via an Interaction with E-cadherin, Is Associated with Activation of Src

**DOI:** 10.1371/journal.pone.0014211

**Published:** 2010-12-03

**Authors:** Siu Man Tsang, Li Liu, Muy-Teck Teh, Ann Wheeler, Richard Grose, Ian R. Hart, David R. Garrod, Farida Fortune, Hong Wan

**Affiliations:** 1 Centre for Clinical and Diagnostic Oral Sciences, Institute of Dentistry, Queen Mary University of London, Barts and The London School of Medicine and Dentistry, London, United Kingdom; 2 Centre for Infectious Disease, Queen Mary University of London, Barts and The London School of Medicine and Dentistry, London, United Kingdom; 3 Imaging Facility, Blizard Institute of Cell and Molecular Sciences, Queen Mary University of London, Barts and The London School of Medicine and Dentistry, London, United Kingdom; 4 Centre for Tumor Biology, Institute of Cancer, Queen Mary University of London, Barts and The London School of Medicine and Dentistry, London, United Kingdom; 5 Faculty of Life Sciences, University of Manchester, Manchester, United Kingdom; 6 King Saud University, Riyadh, Saudi Arabia; University of Birmingham, United Kingdom

## Abstract

**Background:**

Desmoglein 3 (Dsg3), a desmosomal adhesion protein, is expressed in basal and immediate suprabasal layers of skin and across the entire stratified squamous epithelium of oral mucosa. However, increasing evidence suggests that the role of Dsg3 may involve more than just cell-cell adhesion.

**Methodology/Principal Findings:**

To determine possible additional roles of Dsg3 during epithelial cell adhesion we used overexpression of full-length human Dsg3 cDNA, and RNAi-mediated knockdown of this molecule in various epithelial cell types. Overexpression of Dsg3 resulted in a reduced level of E-cadherin but a colocalisation with the E-cadherin-catenin complex of the adherens junctions. Concomitantly these transfected cells exhibited marked migratory capacity and the formation of filopodial protrusions. These latter events are consistent with Src activation and, indeed, Src-specific inhibition reversed these phenotypes. Moreover Dsg3 knockdown, which also reversed the decreased level of E-cadherin, partially blocked Src phosphorylation.

**Conclusions/Significance:**

Our data are consistent with the possibility that Dsg3, as an up-stream regulator of Src activity, helps regulate adherens junction formation.

## Introduction

Desmoglein 3 (Dsg3), a 130 kDa glycoprotein, is a desmosomal cadherin and adhesion molecule. It is one of seven desmosomal cadherins, of two subfamilies, that have been identified in human tissues comprising four desmogleins (Dsg1–4) and three desmocollins (Dsc1–3) [1], [2]. Desmosomes are intercellular junctions that provide strong intercellular links via the desmosome-intermediate filament complex and have a major function in maintaining tissue integrity [1]. In the desmosomal plaque the cytoplasmic domains of the desmosomal cadherins are linked to the intermediate filaments by the armadillo proteins plakoglobin (Pg, also known as γ-catenin) and plakophilins (PP) as well as the plakin family protein desmoplakin (Dp) [3]–[5].

Strong evidence supports a role for Dsg3 in normal desmosomal adhesion [6]–[10]. Thus, many studies have shown that, associated with changes in tissue architecture, the desmosomal cadherins are down-regulated in cancer [11], [12]. Paradoxically, up-regulation of Dsg3 recently has been reported in squamous cell carcinoma and pre-malignant inverted papilloma [13]–[17]. Moreover, RNAi knockdown of Dsg3 in cancer cell lines resulted in inhibition of cell growth, cell migration and invasion [14], suggesting that the role of Dsg3 may be more complex than simply just facilitating normal cell-cell adhesion.

Dsg3 is expressed predominantly in stratified epithelia, including the basal and immediate suprabasal layers of adult epidermis and throughout the stratified layers of oral mucosa [2], [3], [6]. It has been characterised as the auto-antigen of a life-threatening, autoimmune blistering disease, pemphigus vulgaris (PV), in which autoantibodies targeting Dsg3 cause loss of cell-cell adhesion and blister formation in the skin and oral mucosa [18]. Binding of the autoantibodies has been shown to trigger a cascade of intracellular events that may contribute to the pathogenesis of PV, including phosphorylation of Dsg3 and its depletion from desmosomes, induction of apoptosis and modulation of a series of signalling molecules, such as Pg, PKC, p38 MAPK, heat shock protein p27, Src and c-Myc [19]–[28]. Other evidence is suggestive of a possible role for Dsg3 in regulating epidermal differentiation. Thus expression of Dsg3 in mice under the control of the involucrin promoter converted the stratum corneum to a mucous-like phenotype and caused death due to water loss while similar studies using the keratin 1 promoter caused hair thinning, hyperproliferation and abnormal keratin expression [29], [30]. Accordingly Dsg3 is believed to have an important signalling role in addition to its adhesive function.

Recently, we showed an inverse correlation between the expression levels of Dsg3 and cell proliferative capacity in various keratinocyte populations. Cells with low levels of Dsg3 expression (Dsg3-dim) exhibited increased colony forming efficiency and enhanced skin regeneration capability relative to cells with high levels of Dsg3 expression (Dsg3-bright) [31], [32]. Elevated expression of p63, a gene characterised as a stem cell marker, was seen in Dsg3-dim cells compared to Dsg3-bright cells [32], [33]; results again suggesting that Dsg3 might be involved in more than just cell-cell adhesion.

Adherens junctions (AJs) are the other major adhesive intercellular junctions of epithelia. Their adhesion molecules are the classical cadherins, E- and P-cadherin, which associate with the actin cytoskeleton via α-, β- and p120-catenins. It is thought that AJs initiate cell-cell contact while desmosomes reinforce and sustain adhesion [34]–[36]. In tissues desmosomes and adherens junctions appear to be mutually dependent and there is considerable interest in cross-talk between them [35], [37].

Evidence suggests that Dsg3 contributes both to desmosomal adhesion and to regulation of other cellular processes. In order to study the wider functions of Dsg3 we cloned full length human Dsg3 and over-expressed this gene in various epithelial cell lines, including the A431 epidermoid line. Conversely we have knocked down endogenous protein levels in Dsg3 expressing HaCaT cells. Our results reveal novel Dsg3 functions, including complex formation with E-cadherin-catenin, activation of Src and the regulation of E-cadherin-mediated adhesion.

## Results

### Cloning and retroviral expression of the full length human Dsg3

**Figure 1:**
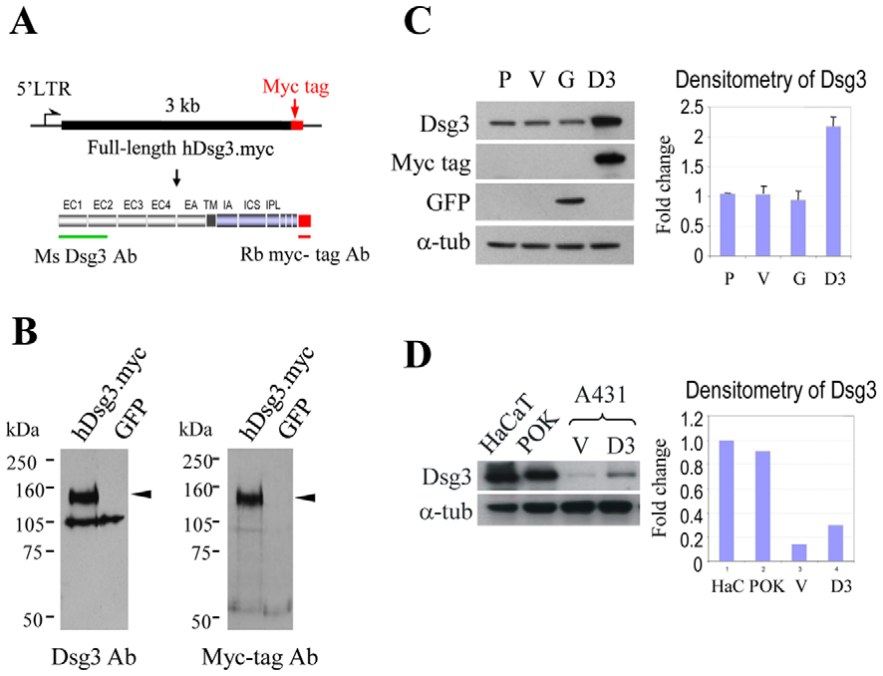
Generation and expression of the retroviral vector containing human Dsg3 cDNA. (A) Schematic diagram of human Dsg3 cDNA tagged with a myc epitope at the C-terminal end (top). Two specific antibodies targeting to EC1-EC2 domains of Dsg3 and the myc tag, respectively, and their recognition sites are shown. (B) Western blotting of the lysates of Cos-1 cells transfected with hDsg3.myc or GFP. Note that both mouse Dsg3 Ab and rabbit myc tag Ab recognised the same band of ∼130 kDa, representing Dsg3. (C) Western blotting and band densitometry analysis of A431 stable cell lines indicated about a >2-fold increase of Dsg3 expression in hDsg3.myc cells as compared with parental and the other two control lines. P. parent; V. empty vector control; G. GFP control; D3. hDsg3.myc. (D) Comparison of Dsg3 expression levels by A431 stable lines, HaCaT (HaC) cells and primary oral keratinocytes (POK). Note that over-expression of Dsg3 in A431 cells did not exceed that of endogenous Dsg3 in primary keratinocytes. The result shown is representative of two independent experiments.

Full length human Dsg3 cDNA, tagged with the c-myc epitope, was cloned into a retroviral vector, pBABE.puro (hDsg3.myc, Fig. 1A). 48 h after transfection of the retroviral vector in Cos-1 cells, Western blotting of cell lysates for hDsg3.myc revealed a distinct band of the correct size (∼130-kDa) that was absent from vGFP control cells (Fig. 1B). Following antibody stripping and re-probing with a rabbit anti-myc antibody, the same sized band was detected (Fig. 1B). A431 cells express moderate levels of endogenous Dsg3 and often have been used as a model to study desmosome function. Thus we generated stable A431 cell lines containing either hDsg3.myc or one of the two controls, vEmpty and vGFP (described in Materials and Methods). Western blotting showed that hDsg3.myc expression in the stable A431 line caused >2-fold increased Dsg3 protein levels relative to the parental, vEmpty and vGFP controls (Fig.1C). We believe such increased expression is not supra- physiological since Dsg3 expression in hDsg3.myc cells remained far below the endogenous levels of Dsg3 found in POK and HaCaT cells (Fig. 1D). Over-expression of hDsg3.myc did not cause cell death while western blotting for several apoptosis-associated proteins showed no differences between the various cell lines (data not shown). There were no changes in cell proliferation, over a two week period, as assessed by cell counting (data not shown).

### Dsg3 co-localises and associates with adherens junction proteins, including E-cadherin

First, we investigated the colocalisation of exogenous human Dsg3 in A431 cells with desmosome proteins such as Dp or another Dsg isoform, Dsg2, by confocal microscopy. Somewhat unexpectedly, we observed limited colocalisation of these proteins. Both Dp and Dsg2 displayed a punctate staining pattern typical of desmosomes while hDsg3.myc showed a diffuse, sinuous, but continuous, membrane distribution. Such limited colocalisation with Dp persisted in cells treated with CSK buffer prior to fixation, a procedure that removes proteins not associated with the cytoskeleton (Data not shown).

**Figure 2:**
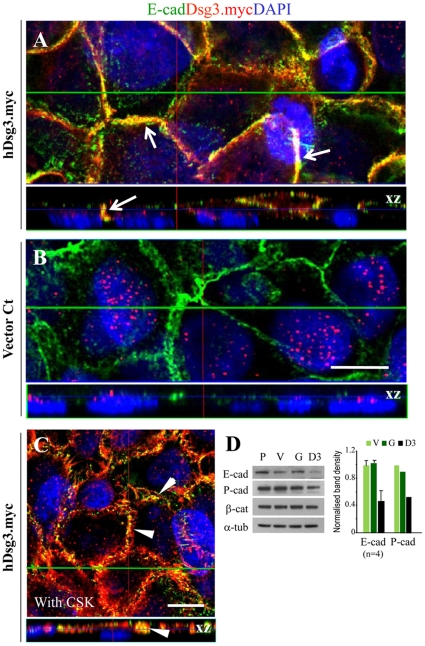
Expression of ectopic hDsg3.myc in A431 cells shows colocalisation with, but down-regulation of, E-cadherin. (A, B) Dual labeling of E-cadherin (green) and myc tag (red) indicates colocalisation of these proteins at the plasma membrane in hDsg3.myc cells (arrows) but not in empty vector control cells that showed single E-cadherin staining. This colocalisation also was seen in cells treated with CSK buffer prior to fixation (C, arrowheads). The xz image panels underneath of each image are the cross section along the green line and show the vertical protein distribution. (D) Western blotting showed reduced expression of the classical E- and P-cadherins, in the hDsg3.myc stable line (D3). P. parent; V. empty vector control; G. GFP control. Quantitation of Western blots, displayed on the right, indicates that E- and P-cadherin showed a 2-fold reduction in D3 cells. Bars, 10 µm.

Next we performed double staining for Dsg3 and E-cadherin. Confocal microscopy revealed substantial colocalisation of hDsg3.myc with E-cadherin, both after routine fixation and after extraction with CSK buffer (Fig. 2A,B,C). Western blotting showed that levels of the classical cadherins E- and P- were reduced, by about 2-fold, in the Dsg3 overexpressing cells (Fig. 2D).

**Figure 3:**
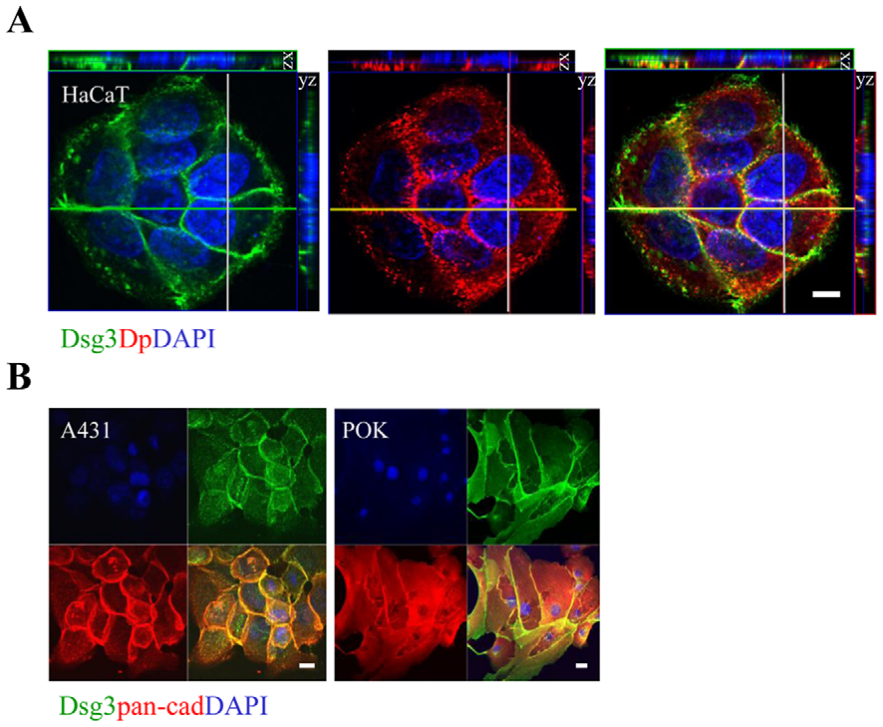
Confocal microscopy of endogenous Dsg3 exhibits partial colocalisation with the desmosomal protein, Dp, but co-localises with the classical cadherins in adherens junctions. (A) Dual labelling for Dsg3 (green) and Dp (red) in HaCaT cells revealed typical punctate staining for Dp but a more continuous membrane staining for Dsg3 with a pattern distinct from that of the desmosomes. (B) A431 and primary oral keratinocytes (POK) were dual labelled for E-cadherin and Dsg3 using a pan-cadherin Ab and mouse Ab 5H10 for Dsg3. Colocalisation of these two proteins is apparent. Bar, 10 µm.

To establish whether endogenous Dsg3 was expressed in a similar distribution pattern we carried out fluorescence confocal microscopy for Dsg3 and Dp in untransfected HaCaT keratinocytes which express high levels of Dsg3 (Fig 1D). Endogenous Dsg3 showed a more continuous cell-peripheral distribution than the punctate pattern usually associated with desmosomes as exemplified by Dp staining (Fig. 3A). Furthermore, staining for Dsg3 and classic cadherins revealed that the endogenous Dsg3 showed extensive colocalisation with cadherins in HaCaTs (data not shown) A431 parental and primary human oral keratinocytes (POK) (Fig. 3B).

**Figure 4:**
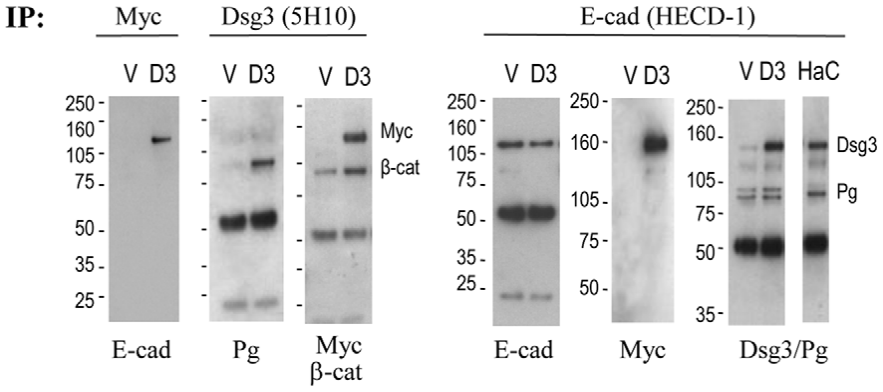
Colocalisation of Dsg3 with E-cadherin-catenin complex. Co-IPs demonstrated that both endogenous and exogenous Dsg3 interacted with the E-cadherin-catenin complex in A431 stable lines (the empty vector control (V) and hDsg3.myc (D3)) as well as in HaCaTs. When pulled down with antibodies against the myc tag or with a mouse Dsg3 Ab 5H10 and blotted for E-cadherin and β-catenin, both proteins were detected in D3 and, to a lesser extent, in V. Conversely, when pulled down with E-cadherin Ab HECD-1 and blotted for Dsg3 or the myc tag and Pg, these proteins were detected in both V and D3 cells. Note that this result also was confirmed in HaCaTs (HaC) where Dsg3 was expressed endogenously (far right blot).

To determine whether colocalisation indicated an interaction between Dsg3 and AJ components we performed co-immunoprecipitation (co-IP) on both A431 vector control and A431-hDsg3.myc cells (Fig. 4). When the lysate of hDsg3.myc cells was pulled down with either anti-myc tag or anti-Dsg3 antibodies and then blotted for E-cadherin or β-catenin, both proteins were detected in the immunoprecipitates (Fig. 4, left). Similar results were obtained using the reverse approach of E-cadherin antibody for the pull down and blotting for myc, Dsg3 and Pg (Fig. 4, right). Similar associations between endogenous Dsg3 and E-cadherin were observed in HaCaT cells (Fig. 4, far right).

### Fluorescene resonance energy transfer (FRET) analysis of Dsg3 and E-cadherin association

**Figure 5:**
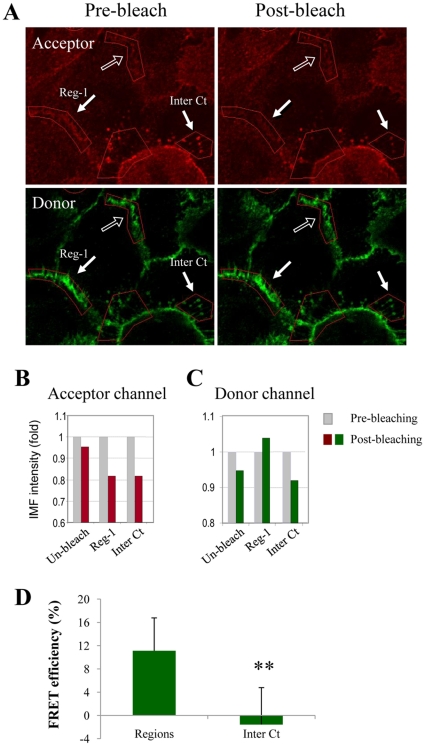
FRET analysis of Dsg3 and E-cadherin association. (A) HaCaT cells were labelled for E-cad with HECD-1 and A488 conjugate and Dsg3 with rabbit Ab and A555 conjugate, respectively. Here arrows designate a peripheral region (Reg-1) with FRET and an intracellular (Inter Ct) region which served as a negative control the open arrow indicates region where no photo-bleaching was performed so that photodamaging was recorded. Bar, 10 µm. (B,C) Numerical quantification of fluorescent intensity in the acceptor and donor channels of the image. (D) Numerical quantification of FRET efficiency (n = 3). ** Statistical significance P<0.01 using Students' T-test.

In order to establish whether the interactions between Dsg3 and E-cadherin that we had detected by confocal microscopy and co-immunoprecipitations were indeed real we used the acceptor photobleaching FRET technique [38]. This procedure is based on the fact that FRET is manifested as an increase in donor fluorescence following photobleaching of the acceptor [38]. As shown in the representative image in Fig. 5A, we used E-cad-A488 as the donor and Dsg3-A555 as the acceptor in HaCaT cells. Region 1 (Reg-1) was selected at the peripheral membrane region of adjacent cells while a control region (Inter Ct) was identified away from this peripheral zone. The histograms show that there was a decline in intensity of fluorescence in the acceptor channel following photo-bleaching (Fig. 5B). In contrast, in the donor channel, fluorescent intensity in the Inter Ct region showed the expected decrease due to the photo damage (as well as for the unbleached region) following a bleach while regions 1 (Reg-1) showed the increase in fluorescence which is indicative of FRET (Fig. 5C). Of 18 peripheral regions analysed in this fashion 7 of them (38%) showed evidence of FRET based on a threshold of >5% increase in IMF intensity relative to the pre-bleached level (Fig. 5D, ** p<0.01). These results indicate that association of Dsg3 and E-cadherin does occur at the cell membrane in HaCaT cells.

### Dsg3 over-expression caused extensive membrane protrusions

**Figure 6 pone-0014211-g006:**
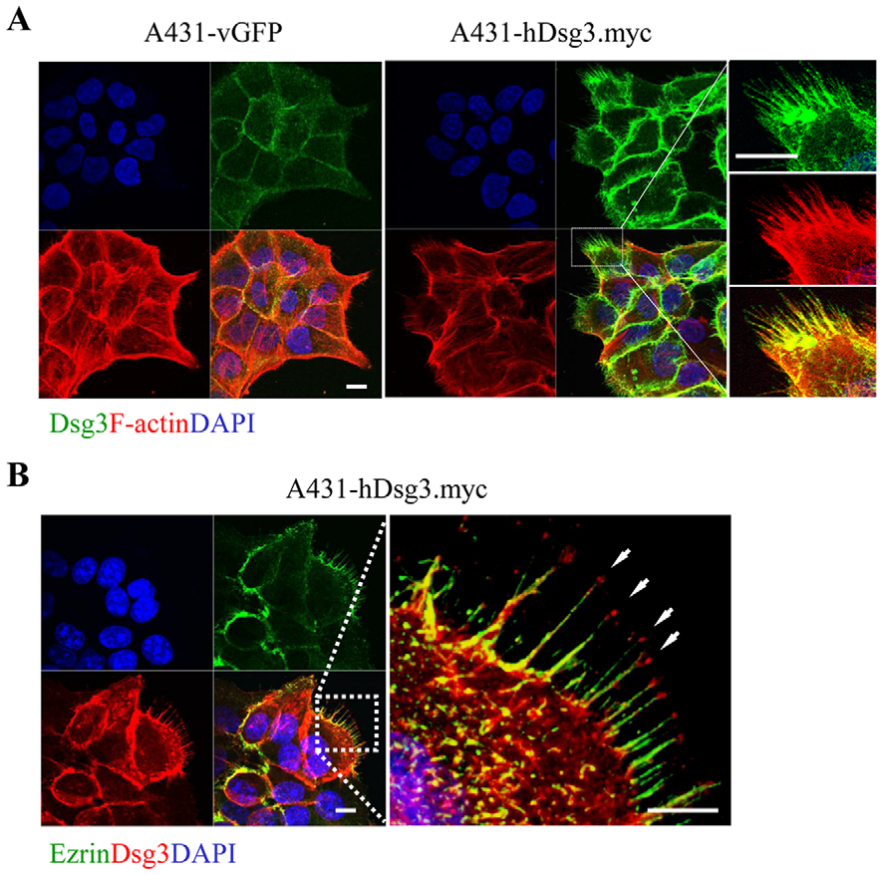
Formation of filopodia by hDsg3.myc cells. (A) Dual labelling of Dsg3 (green) and F-actin (red) in A431 stable lines shows marked filopodium formation in the hDsg3.myc cells. (B) Co-localisation with ezrin (green) established the filopodial nature of these protrusive structures with Dsg3 expression (red) at the tips of each filopodium (arrowheads). Bars, 10 µm.

Interestingly, we noted that transfected hDsg3.myc cells frequently exhibited pronounced membrane protrusions with filopodia formation both at free edges and at cell-cell contacts (Fig. 6). This feature was detailed using immunofluorescent staining for endogenous Dsg3, which highlighted filopodia structure (Fig. 6A), and ezrin localisation (Fig. 6B). Similar morphological changes were observed in other epithelial cells where they appeared to be associated with enhanced cell spreading (Figure S1).

### Dsg3 over-expression accelerates cell migration

**Figure 7:**
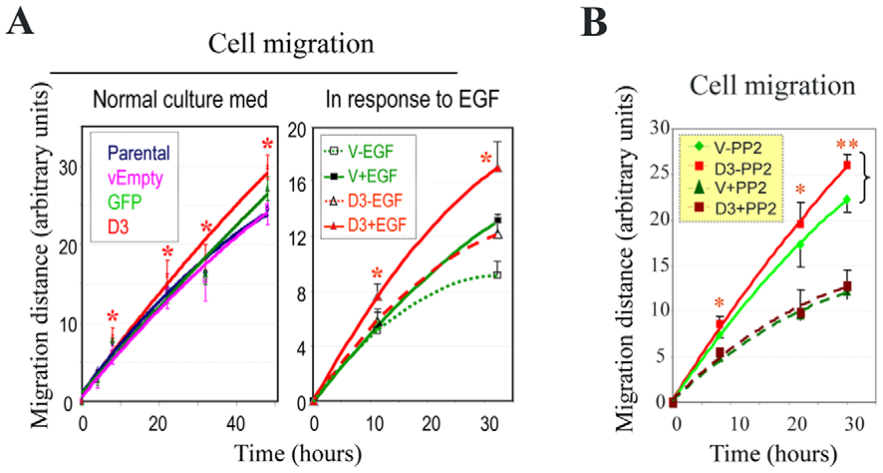
Effect of Dsg3 overexpression on cell migration. (A) Under both normal culture conditions and in the presence of EGF (100 ng/ml) the hDsg3.myc cells were more migratory (p<0.05) than their control counterparts. (B) This increased migratory ability was abrogated almost totally by treating cells with PP2 (10 µM), a Src-specific inhibitor.

The down-regulation of E-cadherin levels and the formation of the extensive protrusions in Dsg3 overexpressing cells reported above were reminiscent of the types of morphological changes observed upon Src activation. This, we hypothesised, might be associated with enhanced motility, especially since such changes were not correlated with any detectable enhanced cell-cell adhesion (Figure S2). Consequently we monitored the rate of cell migration using a Scratch wound assay. As seen in Figure 7A, there was an increased migration by A431-hDsg3.myc cells compared to all controls both under normal culture conditions (DMEM plus 10% FCS) and in the presence of EGF (100 ng/ml). This enhanced migration was blocked completely in the presence of PP2, a specific inhibitor of Src (Fig. 7B), that has been reported to be able to restore the E-cadherin-mediated cell adhesion in human cancer cells and to reduce cancer metastasis [39].

### Dsg3 forms a complex with Src and regulates its activity

**Figure 8:**
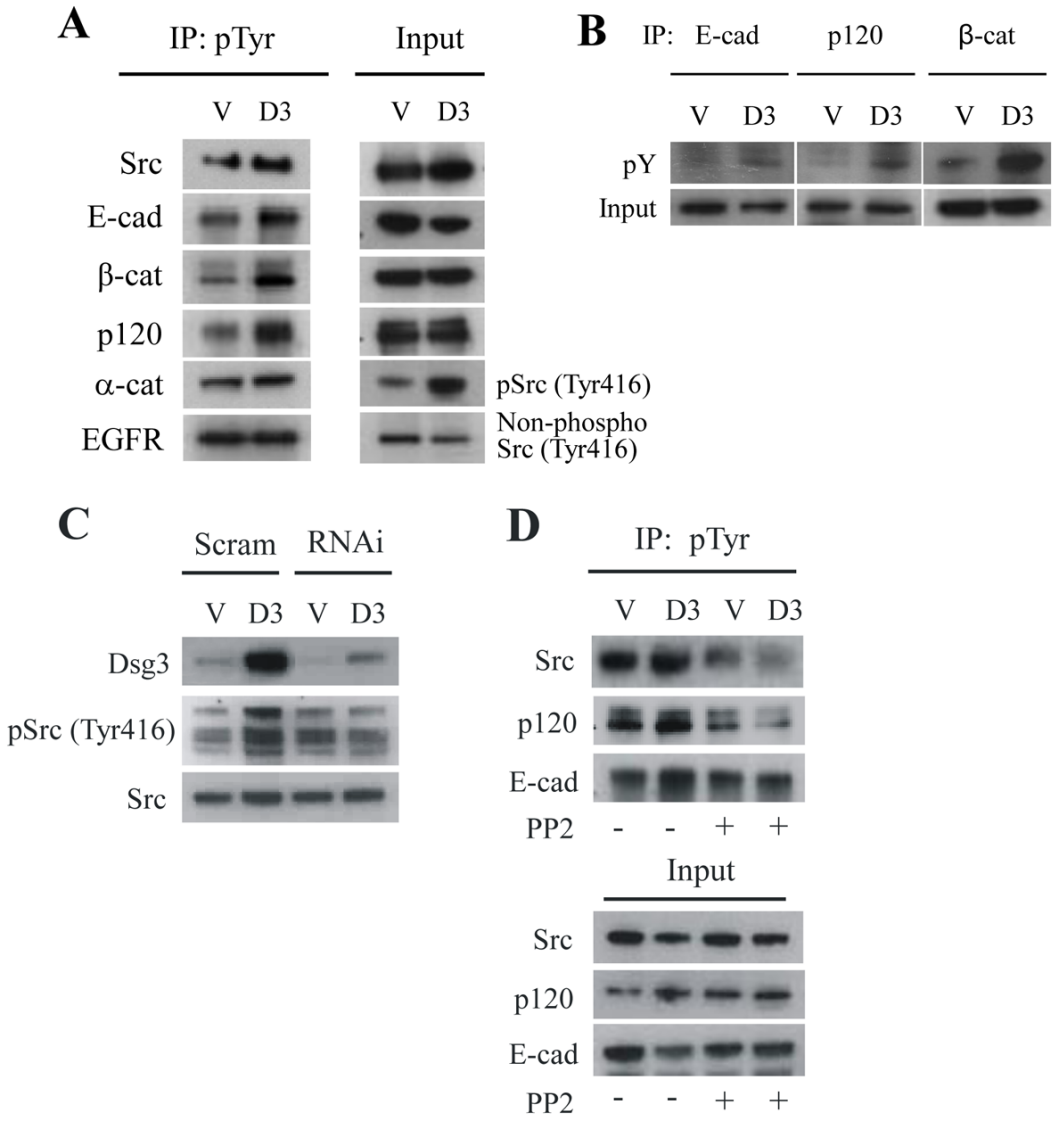
Over-expression of Dsg3 caused activation of Src and increased phosphorylation of AJ proteins. (A) Western blotting of immunoprecipitates, pulled down with anti-phospho-Tyr antibody from lysates of A431 stable lines treated with Ca-free medium for 1 hour prior to a calcium switch for 5 hours. Levels of tyrosine phosphorylation of Src and E-cadherin junction proteins, including α-, β- and 120 catenins as well as E-cadherin, were increased in cells over-expressing Dsg3 (D3) compared with those in the vector control cells (V). EGFR served as the negative control. The results shown are representative from four independent experiments. (B) Similar results were seen with reverse procedures where immunoprecipitations were with anti-E-cadherin, -p120 or -β-catenin antibodies, respectively, followed by blotting for phospho- Tyr. (C) Western blotting of A431 cells transfected with scrambled (Scram) or Dsg3 siRNA (RNAi) for 2 days. Overexpression of Dsg 3 was associated with increased expression of phosphorylated Src but this increase was abrogated by knockdown of Dsg 3. (D) Presence of the Src-specific inhibitor PP2 (10 µM) blocked both the increased phosphorylation of p120 and E-cadherin and the reduced level of total E-cadherin that was associated with hDsg3.myc cells (D3) compared to vector control cells (V).

Activation of Src, with tyrosine phosphorylation of β- and p120-catenins, has been documented in calcium-induced keratinocyte cell-cell adhesion [40], [41]. Therefore we sought to examine adhesion-dependent phosphorylation of Src and its AJ protein substrates in A431 cells. Comparison between hDsg3.myc and control cells, in which cell-cell adhesion had been calcium-induced for 5 hours, showed that phosphorylation of Src and the AJ proteins E-cadherin and the catenins was greater in the hDsg3.myc cells (Fig. 8A,B). These results suggested that Dsg3 can regulate Src activity.

When RNAi was used to knock-down Dsg3 in hDsg3.myc cells the increase of phospho-Src levels (Fig. 8C) was reduced suggesting that Dsg3 may act upstream in the Src pathway. Calcium-induced A431 Dsg3.myc cell adhesion was utilised in the presence of the Src-specific inhibitor, PP2 (10 µM, [42], [43]). This resulted in a substantial inhibition of Src activation as well as a reduction of the phosphorylation of E-cadherin and p120 (Fig. 8D). Equally PP2 blocked the down-regulation of total E-cadherin levels that always was associated with Dsg3 overexpression (Fig. 8D, Input).

Collectively the changes in phenotype observed in A431-hDsg3 myc cells, i.e. increased phosphorylation of E-cadherin-catenin complexes, pronounced membrane protrusions and augmented cell motility, are consistent with the possibility that Dsg3 is associated with Src activation [44], [45].

## Discussion

Our studies have utilised a full length human Dsg3 cDNA which allowed the generation of a human cell line stably over-expressing this gene. Subsequent studies with this cell line suggested a novel association and function for the desmosomal cadherin Dsg3. Thus we were able to show that Dsg3 brought about a reduction in E-cadherin levels while it formed a complex with E-cadherin and other adherens junction proteins. Moreover, Dsg3 appeared to play a critical role in regulating E-cadherin-Src signalling, being a positive regulator of its activity, with consequences for formation of membrane projections and cell migratory capacity. These results were shown not to be simply a consequence of overexpression through the examination of endogenous proteins and the utilisation of RNAi in lines which had high endogenous levels of the Dsg3 protein.

Initially we noted that Dsg3 displayed continuous membrane distribution rather than the punctate staining pattern expected for desmosomal proteins (Fig. 2). There have been several reports that PV autoantibodies bind to the inter-desmosomal keratinocyte membrane but, since such antibodies are not specific for Dsg3, it could not safely be concluded that this binding represented Dsg3 distribution [46]–[49]. It is contended that there are two pools of Dsg3 at the plasma membrane, one in desmosomes and another which is extra-desmosomal [28], [50], [51], though, the actual nature of this extra-demosomal pool remains unclear. Colocalisation of desmosomal components with AJ proteins has been reported in the intercalated discs of cardiac muscle [52]. However it was unclear, as it is in our studies, whether the interaction between Dsg3 and E-cadherin in the complex was direct or indirect. We could speculate that it may be indirect, since several candidate junctional proteins, including Pg, p120 catenin and p0071 catenin, have been shown to bind both classical and desmosomal cadherins [50], [53], [54] though it also is true that our results demonstrating FRET speak for a more direct interaction. Our observations conflict somewhat with an earlier report, which was based solely on immunoprecipitation from radio-labelled human keratinocytes, suggesting that Dsg3 co-precipitates with Pg only [55]. We have used a variety of different techniques to confirm the association with E-cadherin but we are unable to confirm this earlier observation [55]. Such a discrepancy might well be due to different culture conditions and/or different detection methodologies.

Phosphotyrosine signalling mediated by Src family kinases is one of the pathways which is activated upon E-cadherin mediated cell-cell adhesion [40], [41], [44], [56]. However, evidence for both a positive and a negative contribution of such signalling events in affecting cadherin-catenin complex stability and junctional integrity has been reported [40], [41], [44], [45], [57]. Based on our studies we speculate that Dsg3 may be a key player in Src signalling during formation of cell-cell adhesions. Over-expression of Dsg3 in A431 cells elicited a phenotype similar to that of cells with aberrantly increased Src activity [44]. The phenotype included: 1) suppression of classical cadherins; 2) alteration of E-cadherin distribution at the plasma membrane; 3) elevated tyrosine phosphorylation of E-cadherin-catenin complexes; 4) augmented cell motility; 5) pronounced membrane protrusions (Fig. 5–7). Such changes are consistent with the observation that in squamous cell carcinoma (head and neck, and lung) and inverted papilloma augmented expression of Dsg3 has been reported [13]–[15], [17], [58]. Our data suggest that Src activation in hDsg3.myc cells was independent of EGFR in our culture conditions, since no changes were detected either in levels of protein expression or in its phosphorylation status [43] (Fig. 8A). Tyrosine phosphorylation of E-cadherin leads to its ubiquitination and lysosomal degradation [59] while a role for p120 in cell motility, via modulation of the activity of Rho-family GTPases, as well as in cell-cell adhesion has been documented [60]. Thus over-activation or ablation of these signalling pathways may have an impact on these cellular processes. It seems likely that tightly controlled physiological levels of Src signals in AJs are critical for the assembly and maintenance of intercellular adhesion, while dysregulation results in abnormalities of cell junction formation and migratory behaviour that might lead to significant pathology. Our results suggest that Dsg3, rather unexpectedly, might contribute to these adherens junction-mediated events.

## Materials and Methods

### Generation of retroviral constructs

Human Dsg3 cDNA was cloned by PCR from extracted total RNA of primary human epidermal keratinocytes as described previously [33] using primers derived from the published sequence (Accession: NM_001944.1)(http://www.ncbi.nlm.nih.gov/nuccore/4503404). The full-length cDNA was assembled from two overlapping clones, generated by digestion of PCR products containing the Sal I and Bam HI restriction sites, and a small region of synthesised nucleotides (2977–2997) plus the c-Myc tag and a stop codon and cloned into the retroviral vector, pBABE.puro. The original multi-cloning site (MCS) of pBABE.puro first was rearranged in the order of *EcoR I-SnaB I-Sal I-Bam HI-Bgl II* so that the full length cDNA ultimately was subcloned into *EcoR I/Bgl II* restriction sites to create hDsg3.myc. The full-length cDNA was verified by extensive sequencing to ensure that no error had been introduced.

### Antibodies

The following mouse monoclonal and rabbit polyclonal Abs were used: 5H10, mouse Ab against Dsg3 (gift from Professor M Amagai); AHP319, rabbit Ab against Dsg3 (Serotec); 10G11, mouse anti-human Dsg2 (Progen); Dsc3-U114, mouse anti-human Dsc3 (Progen); Pab to Dsc2, rabbit Ab to Dsc2a and Dsc2b (Progen); 115F, mouse Ab against Dp (in house); AHP320, rabbit Ab against Dp (Serotec); PG5.1, mouse Ab against Pg (Millipore); Mab to PKP2 (2a+2b) multi-epitope cocktail (Progen); HECD-1, mouse anti-E-cadherin (Abcam); mouse anti-P-Cadherin (Zymed laboratories Inc.); CH-19, rabbit anti-pan Cadherin (Zymed laboratory); 6F9, mouse anti-β-catenin (Sigma); mouse anti-p120 catenin (BD Transduction Laboratories); rabbit polyclonal to p120 (H-90) (Santa Cruz Biotechnology); rabbit polyclonal to Myc tag (Abcam and Novus Biologicals, Inc); rabbit pAb to GFP (Abcam); Src (32G6) rabbit mAb, phospho-Src family (Tyr416) and non-phospho-Src (Tyr416) mouse mAb (Cell Signaling); rabbit anti-phospho-Tyrosine p-Tyr (PY350) (Santa Cruz Biotechnology); rabbit anti beta actin-loading control ab8227 (Abcam); DM1A, mouse monoclonal to alpha Tubulin-loading control ab7291 (Abcam); Secondary Abs were Alexa Fluor 488/568 conjugated goat anti-mouse IgG and Alexa Fluor 488/568 conjugated goat anti-rabbit IgG (Invitrogen).

### Retroviral transfection and transduction with the hDsg3.myc construct

Cos-1, HaCaT and A431 cell lines were cultured in DMEM containing 10% FCS. Transient transfection was conducted using FuGENE Transfection Reagents (Roche) following manufacturer's instructions. The retroviral transfection and transduction were carried out as described elsewhere [61], generating ∼40% transient transfection efficiency. For generation of stable cell stocks with over-expression of human Dsg3 (hDsg3.myc), or the empty vector (vEmpty) and GFP (vGFP) controls, A431 cells were spin infected with retroviral-conditioned medium for 1 hour and then subjected to drug selection 2 days later and maintained in 1 µg/ml puromycin consistently for 2 weeks (during which period of time the majority of cells died out and only a few colonies survived). These colonies were pooled and polyclonal cell populations were transferred to 0.25 µg/ml puromycin and either maintained in culture or frozen down as cell stocks (stable lines). The experiments were carried out on cells where drug removal had been for over 2 weeks. The individual clones of cells with overexpression of hDsg3.myc were generated by limited dilution and cloned cells were then propagated prior to be used in the study. We found that the expression of hDsg3.myc in A431 cells was stable for over 3 months during which time all experiments were performed.

### Immunofluorescence and confocal microscopy

Cultured cells grown on coverslips were fixed and permeabilised with either ice-cold 1∶1 methanol/acetone for 10 minutes or 4% formaldehyde for 10 minutes and then 0.1% Triton X-100 for 5 minutes, respectively, before immunofluorescent staining with the indicated antibodies. All the mounted coverslips were examined with a Zeiss LSM 510s laser scanning confocal microscope using a 63x 1.4NA Plan Apochromat objective a Leica DM5000 epifluorescence microscope. For quantitative analysis of peripheral fluorescence intensity for Dsg2 and Dsg3 staining, a profile tool of Zeiss confocal software was used. For analysis of membrane protrusions, positive cells with filopodia were counted (blind) from more than 4 fields at x63 objective and the percentage of positive cells was calculated. In this case more than 400 cells were scored in each group.

### Scratch wound assay

A431 cells were seeded at a confluent density in 6-well plates. A scratch wound was made the next day using a plastic pipette tip. The migration of the epithelial cell sheet was monitored at three marked areas along the line of the wound. Snapshots of each marked area were taken over a period of time. Wound gap closure was measured using Adobe Photoshop and all values were recorded and calculated in Microsoft Excel. The cell migration distance at each marked area for each time point was calculated by subtracting the value from the 0 hour time point and then dividing by two. The data presented are the mean±sd from the three marked points for each sample (Exp n>3). In experiments with EGF, cells were subjected to serum starvation overnight before scratch wounding. Then wound closure was monitored in the presence or absence of EGF (100 ng/ml).

### FRET analysis

FRET was measured, using acceptor photobleaching [38] with a Zeiss inverted 510 confocal and a 63x NA 1.4 oil immersion Plan Apochromat objective and a software zoom of 2. HaCaT cells were grown on coverslips as described previously. E-cadherin and Dsg3 double staining was performed using HECD-1 combined with FAb fragments of Alexa Fluor 488 nm secondary antibody conjugate and rabbit anti-Dsg3 and Alexa Fluor 555 nm conjugate, respectively. FRET was carried out using two laser lines; firstly the 488 nm line of an Argon laser (Lasos) set at 6.1A using 5% laser power and a pinhole of 1. Secondly a 543 nm line from a HeNe laser (Lasos) using 100% laser power with a pinhole of 1. The settings of the PMTs used for collecting data from the Acceptor fluorescence and FRET channels were identical in terms of detector gain and offset and filters use (for the Donor this is 505–550 nm Band Pass filter, for acceptor and FRET a 560 nm Long Pass filter). Photobleaching of the acceptor channel was carried out using 543 nm excitation wavelength at 100% with a dwell time of 40 µs using 150 iterations to ensure reduction of the fluorescent intensity in the region of interest by at least 20%. As a negative control, an intracellular region was collected and photobleached in each image. In addition, a peripheral region without photobleaching was collected for calculation of photodamage in each image. The FRET was calculated as the percentage of increase of donor fluorescence (A488) after photobleaching of acceptor (A555), in the regions of interest of cell junctions by that following formula (FRET  =  [(*Ipost* + photodamage)/*Ipre* – 1]*100%). We corrected for loss of fluorescence in the donor channel by measuring the intensity of donor fluorescence before and after photobleaching in a region which is not photobleached. Regions with FRET greater than 5% (threshold) were considered to be positive. 18 peripheral regions of cells were analysed in four areas and FRET is presented as mean±sd. Control images for the AbFRET experiments to show the level of acceptor fluorescence at FRET-excitation, and acceptor fluorescence at optimum excitation for the A555 chromophore are provided in Figure S3.

### Transient Dsg3 siRNA transfection

A siRNA sequence specific for human Dsg3 mRNA, corresponding to nucleotides 620 to 640 of the respective coding region (Accession: NM_001944.1) (AAATGCCACAGATGCAGATGA) was designed and this sequence was subjected to a BLAST database search prior to being synthesised by Dharmacon Research (USA). The scrambled control, a randomised version of Dsg3 siRNA sequence (AACGATGATACATGACACGAG), was also synthesised by the same company. The transfection procedures were described previously [62].

### Calcium switch assay

HaCaT cells were seeded at confluence density and grown in low calcium medium (EpiLife, Cascade Biologics) until confluent (2–3 d). They were then transferred into normal calcium medium (2 mM in Epilife) for different times according to the individual experiments. For immunofluorescent staining, the siRNA transfected cells were subject to the calcium switch for 1, 4, 8 and 24 hours prior to fixation. In co-IP experiments, cell grown in 100 mm Petri dishes were subject to calcium switch for 0, 2, 8 and 48 hours prior to cell lysis. For protein phosphorylation analysis, cells were grown in normal medium, to reach sufficient confluence, before treatment with calcium-free medium (DMEM supplemented with 10% decalcified FCS plus 3 mM EGTA) for 1 hour followed by a calcium switch for different times according to specific experiments.

### Co-immunoprecipitation and Western blotting

Protein extraction and Western blotting were carried out as described previously [31], [32], [62]. For co-IP, cells were washed with ice-cold PBS, then lysed in ice cold RIPA buffer (Upstate) containing a protease-inhibitor cocktail (Calbiochem) for 10 minutes at 4°C. Lysates were clarified by microcentrifugation. Protein concentration was determined by Bio-Rad DC protein assay. A certain amount (normally 0.5–1 mg) of total protein was used for IP with Abs coupled with Dynabeads (Invitrogen) for 3 hours before adding into the cell lysates and incubation overnight at 4°C with rotation. Immunoprecipitates were washed thoroughly prior to resuspension in 2x Laemmli sample buffer and boiled for 3 minutes. Aliquots of the denatured proteins were separated by SDS-PAGE and processed for Western blotting.

## Supporting Information

Figure S1 Expression of Dsg3 caused faster and wider spreading of 293T cells. Cells were transfected transiently with either the pBABE-GFP or -hDsg3.myc construct for two days before harvesting with trypsin. Cells then were seeded at low density onto coverslips without coating and fixed at various time points prior to immunostaining for the indicated proteins. Images were analysed in ImageJ software and an area of over 20 positively transfected cells in each group (pixel no per cell), from 4 arbitrary fields, was scored and presented as mean±sd (*p<0.002).
(9.72 MB TIF)

Figure S2 Over-expression of Dsg3 failed to enhance cell-cell adhesion. Cells were grown to confluence before being treated with 2.4 units/ml dispase for 20 minutes to detach the epithelial sheets. The epithelial sheets were washed with PBS, twice gently, before being subjected to mechanical stress by pipetting three-five times with 1-ml blue tips. The epithelial fragments were quantitated by ImageJ and presented as the mean+sd (Exp n = 6). The presented data were pooled from three independent experiments.
(9.91 MB TIF)

Figure S3 Control images for the AbFRET experiments, showing the level of bleed through into the FRET channel of cells labelled with the Donor alone and Acceptor alone. The FRET channel is shown using the Spectrum look up table from Image J.
(9.53 MB TIF)

## References

[1] Garrod D, Chidgey M (2008). Desmosome structure, composition and function.. Biochim Biophys Acta.

[2] Getsios S, Huen AC, Green KJ (2004). Working out the strength and flexibility of desmosomes.. Nat Rev Mol Cell Biol.

[3] Garrod DR, Merritt AJ, Nie Z (2002). Desmosomal cadherins.. Curr Opin Cell Biol.

[4] Green KJ, Gaudry CA (2000). Are desmosomes more than tethers for intermediate filaments?. Nat Rev Mol Cell Biol.

[5] Gumbiner BM (1996). Cell adhesion: the molecular basis of tissue architecture and morphogenesis.. Cell.

[6] Amagai M, Koch PJ, Nishikawa T, Stanley JR (1996). Pemphigus vulgaris antigen (desmoglein 3) is localized in the lower epidermis, the site of blister formation in patients.. J Invest Dermatol.

[7] Koch PJ, Mahoney MG, Ishikawa H, Pulkkinen L, Uitto J (1997). Targeted disruption of the pemphigus vulgaris antigen (desmoglein 3) gene in mice causes loss of keratinocyte cell adhesion with a phenotype similar to pemphigus vulgaris.. J Cell Biol.

[8] Koch PJ, Mahoney MG, Cotsarelis G, Rothenberger K, Lavker RM (1998). Desmoglein 3 anchors telogen hair in the follicle.. J Cell Sci.

[9] Pulkkinen L, Choi YW, Simpson A, Montagutelli X, Sundberg J (2002). Loss of cell adhesion in Dsg3bal-Pas mice with homozygous deletion mutation (2079del14) in the desmoglein 3 gene.. J Invest Dermatol.

[10] Shimizu A, Ishiko A, Ota T, Tsunoda K, Koyasu S (2002). Ultrastructural changes in mice actively producing antibodies to desmoglein 3 parallel those in patients with pemphigus vulgaris.. Arch Dermatol Res.

[11] Fukuoka J, Dracheva T, Shih JH, Hewitt SM, Fujii T (2007). Desmoglein 3 as a prognostic factor in lung cancer.. Hum Pathol.

[12] Shinohara M, Hiraki A, Ikebe T, Nakamura S, Kurahara S (1998). Immunohistochemical study of desmosomes in oral squamous cell carcinoma: correlation with cytokeratin and E-cadherin staining, and with tumour behaviour.. J Pathol.

[13] Liu ZQ, Tian YQ, Ma FR, Zhu L, Hu YF (2007). [Expression of desmoglein 3 in nasopharyngeal carcinoma: research of 22 cases].. Zhonghua Yi Xue Za Zhi.

[14] Chen YJ, Chang JT, Lee L, Wang HM, Liao CT (2007). DSG3 is overexpressed in head neck cancer and is a potential molecular target for inhibition of oncogenesis.. Oncogene.

[15] Pittella F, Katsube K, Takemura T, Hashimoto T, Kawano T (2001). Perinuclear and cytoplasmic distribution of desmoglein in esophageal squamous cell carcinomas.. Pathol Res Pract.

[16] Huang CC, Lee TJ, Chang PH, Lee YS, Chuang CC (2009). Desmoglein 3 is overexpressed in inverted papilloma and squamous cell carcinoma of sinonasal cavity.. Laryngoscope.

[17] Savci-Heijink CD, Kosari F, Aubry MC, Caron BL, Sun Z (2009). The role of desmoglein-3 in the diagnosis of squamous cell carcinoma of the lung.. Am J Pathol.

[18] Amagai M, Klaus-Kovtun V, Stanley JR (1991). Autoantibodies against a novel epithelial cadherin in pemphigus vulgaris, a disease of cell adhesion.. Cell.

[19] Aoyama Y, Owada MK, Kitajima Y (1999). A pathogenic autoantibody, pemphigus vulgaris-IgG, induces phosphorylation of desmoglein 3, and its dissociation from plakoglobin in cultured keratinocytes.. Eur J Immunol.

[20] Berkowitz P, Hu P, Liu Z, Diaz LA, Enghild JJ (2005). Desmosome signaling. Inhibition of p38MAPK prevents pemphigus vulgaris IgG-induced cytoskeleton reorganization.. J Biol Chem.

[21] Berkowitz P, Hu P, Warren S, Liu Z, Diaz LA (2006). p38MAPK inhibition prevents disease in pemphigus vulgaris mice.. Proc Natl Acad Sci U S A.

[22] Caldelari R, de Bruin A, Baumann D, Suter MM, Bierkamp C (2001). A central role for the armadillo protein plakoglobin in the autoimmune disease pemphigus vulgaris.. J Cell Biol.

[23] Kawasaki Y, Aoyama Y, Tsunoda K, Amagai M, Kitajima Y (2006). Pathogenic monoclonal antibody against desmoglein 3 augments desmoglein 3 and p38 MAPK phosphorylation in human squamous carcinoma cell line.. Autoimmunity.

[24] Kitajima Y, Aoyama Y, Seishima M (1999). Transmembrane signaling for adhesive regulation of desmosomes and hemidesmosomes, and for cell-cell datachment induced by pemphigus IgG in cultured keratinocytes: involvement of protein kinase C.. J Investig Dermatol Symp Proc.

[25] Osada K, Seishima M, Kitajima Y (1997). Pemphigus IgG activates and translocates protein kinase C from the cytosol to the particulate/cytoskeleton fractions in human keratinocytes.. J Invest Dermatol.

[26] Pelacho B, Natal C, Espana A, Sanchez-Carpintero I, Iraburu MJ (2004). Pemphigus vulgaris autoantibodies induce apoptosis in HaCaT keratinocytes.. FEBS Lett.

[27] Chernyavsky AI, Arredondo J, Kitajima Y, Sato-Nagai M, Grando SA (2007). Desmoglein versus non-desmoglein signaling in pemphigus acantholysis: characterization of novel signaling pathways downstream of pemphigus vulgaris antigens.. J Biol Chem.

[28] Williamson L, Raess NA, Caldelari R, Zakher A, de Bruin A (2006). Pemphigus vulgaris identifies plakoglobin as key suppressor of c-Myc in the skin.. EMBO J.

[29] Elias PM, Matsuyoshi N, Wu H, Lin C, Wang ZH (2001). Desmoglein isoform distribution affects stratum corneum structure and function.. J Cell Biol.

[30] Merritt AJ, Berika MY, Zhai W, Kirk SE, Ji B (2002). Suprabasal desmoglein 3 expression in the epidermis of transgenic mice results in hyperproliferation and abnormal differentiation.. Mol Cell Biol.

[31] Wan H, Stone MG, Simpson C, Reynolds LE, Marshall JF (2003). Desmosomal proteins, including desmoglein 3, serve as novel negative markers for epidermal stem cell-containing population of keratinocytes.. J Cell Sci.

[32] Wan H, Yuan M, Simpson C, Allen K, Gavins FN (2007). Stem/progenitor cell-like properties of desmoglein 3dim cells in primary and immortalized keratinocyte lines.. Stem Cells.

[33] Koster MI, Kim S, Roop DR (2005). P63 deficiency: a failure of lineage commitment or stem cell maintenance?. J Investig Dermatol Symp Proc.

[34] Gumbiner B, Stevenson B, Grimaldi A (1988). The role of the cell adhesion molecule uvomorulin in the formation and maintenance of the epithelial junctional complex.. J Cell Biol.

[35] Vasioukhin V, Bauer C, Yin M, Fuchs E (2000). Directed actin polymerization is the driving force for epithelial cell-cell adhesion.. Cell.

[36] Yin T, Green KJ (2004). Regulation of desmosome assembly and adhesion.. Semin Cell Dev Biol.

[37] Lewis JE, Wahl JK, III, Sass KM, Jensen PJ, Johnson KR (1997). Cross-talk between adherens junctions and desmosomes depends on plakoglobin.. J Cell Biol.

[38] Karpova TS, Baumann CT, He L, Wu X, Grammer A (2003). Fluorescence resonance energy transfer from cyan to yellow fluorescent protein detected by acceptor photobleaching using confocal microscopy and a single laser.. J Microsc.

[39] Nam JS, Ino Y, Sakamoto M, Hirohashi S (2002). Src family kinase inhibitor PP2 restores the E-cadherin/catenin cell adhesion system in human cancer cells and reduces cancer metastasis.. Clin Cancer Res.

[40] Calautti E, Cabodi S, Stein PL, Hatzfeld M, Kedersha N (1998). Tyrosine phosphorylation and src family kinases control keratinocyte cell-cell adhesion.. J Cell Biol.

[41] Calautti E, Grossi M, Mammucari C, Aoyama Y, Pirro M (2002). Fyn tyrosine kinase is a downstream mediator of Rho/PRK2 function in keratinocyte cell-cell adhesion.. J Cell Biol.

[42] Yin T, Getsios S, Caldelari R, Kowalczyk AP, Muller EJ (2005). Plakoglobin suppresses keratinocyte motility through both cell-cell adhesion-dependent and -independent mechanisms.. Proc Natl Acad Sci U S A.

[43] Heupel WM, Engerer P, Schmidt E, Waschke J (2009). Pemphigus vulgaris IgG cause loss of desmoglein-mediated adhesion and keratinocyte dissociation independent of epidermal growth factor receptor.. Am J Pathol.

[44] McLachlan RW, Yap AS (2007). Not so simple: the complexity of phosphotyrosine signaling at cadherin adhesive contacts.. J Mol Med.

[45] Owens DW, McLean GW, Wyke AW, Paraskeva C, Parkinson EK (2000). The catalytic activity of the Src family kinases is required to disrupt cadherin-dependent cell-cell contacts.. Mol Biol Cell.

[46] Sato M, Aoyama Y, Kitajima Y (2000). Assembly pathway of desmoglein 3 to desmosomes and its perturbation by pemphigus vulgaris-IgG in cultured keratinocytes, as revealed by time-lapsed labeling immunoelectron microscopy.. Lab Invest.

[47] Suter MM, Wilkinson JE, Dougherty EP, Lewis RM (1990). Ultrastructural localization of pemphigus vulgaris antigen on canine keratinocytes in vivo and in vitro.. Am J Vet Res.

[48] Bedane C, Prost C, Thomine E, Intrator L, Joly P (1996). Binding of autoantibodies is not restricted to desmosomes in pemphigus vulgaris: comparison of 14 cases of pemphigus vulgaris and 10 cases of pemphigus foliaceus studied by western immunoblot and immunoelectron microscopy.. Arch Dermatol Res.

[49] Takahashi H, Wada T, Matsuo S, Iwatsuki K, Iizuka H (1995). A case of bullous pemphigoid with antibodies against intercellular 130 kd antigen.. J Dermatol.

[50] Kanno M, Isa Y, Aoyama Y, Yamamoto Y, Nagai M (2008). P120-catenin is a novel desmoglein 3 interacting partner: identification of the p120-catenin association site of desmoglein 3.. Exp Cell Res.

[51] Delva E, Jennings JM, Calkins CC, Kottke MD, Faundez V (2008). Pemphigus vulgaris IgG-induced desmoglein-3 endocytosis and desmosomal disassembly are mediated by a clathrin- and dynamin-independent mechanism.. J Biol Chem.

[52] Borrmann CM, Grund C, Kuhn C, Hofmann I, Pieperhoff S (2006). The area composita of adhering junctions connecting heart muscle cells of vertebrates. II. Colocalizations of desmosomal and fascia adhaerens molecules in the intercalated disk.. Eur J Cell Biol.

[53] Setzer SV, Calkins CC, Garner J, Summers S, Green KJ (2004). Comparative analysis of armadillo family proteins in the regulation of a431 epithelial cell junction assembly, adhesion and migration.. J Invest Dermatol.

[54] Roh JY, Stanley JR (1995). Plakoglobin binding by human Dsg3 (pemphigus vulgaris antigen) in keratinocytes requires the cadherin-like intracytoplasmic segment.. J Invest Dermatol.

[55] Plott RT, Amagai M, Udey MC, Stanley JR (1994). Pemphigus vulgaris antigen lacks biochemical properties characteristic of classical cadherins.. J Invest Dermatol.

[56] McLachlan RW, Kraemer A, Helwani FM, Kovacs EM, Yap AS (2007). E-cadherin adhesion activates c-Src signaling at cell-cell contacts.. Mol Biol Cell.

[57] Ezaki T, Guo RJ, Li H, Reynolds AB, Lynch JP (2007). The homeodomain transcription factors Cdx1 and Cdx2 induce E-cadherin adhesion activity by reducing beta- and p120-catenin tyrosine phosphorylation.. Am J Physiol Gastrointest Liver Physiol.

[58] Huang CC, Lee TJ, Chang PH, Lee YS, Chuang CC (2009). Desmoglein 3 is overexpressed in inverted papilloma and squamous cell carcinoma of sinonasal cavity.. Laryngoscope.

[59] Shen Y, Hirsch DS, Sasiela CA, Wu WJ (2008). Cdc42 regulates E-cadherin ubiquitination and degradation through an epidermal growth factor receptor to Src-mediated pathway.. J Biol Chem.

[60] Grosheva I, Shtutman M, Elbaum M, Bershadsky AD (2001). p120 catenin affects cell motility via modulation of activity of Rho-family GTPases: a link between cell-cell contact formation and regulation of cell locomotion.. J Cell Sci.

[61] Soneoka Y, Cannon PM, Ramsdale EE, Griffiths JC, Romano G (1995). A transient three-plasmid expression system for the production of high titer retroviral vectors.. Nucleic Acids Res.

[62] Wan H, South AP, Hart IR (2007). Increased keratinocyte proliferation initiated through downregulation of desmoplakin by RNA interference.. Exp Cell Res.

