# Supplementary figures and images for: Desmoglein 3, via an Interaction with E-cadherin, Is Associated with Activation of Src
Source: PLoS One. 2010 Dec 3;5(12):e14211. doi: 10.1371/journal.pone.0014211 (PMC2997060; doi:10.1371/journal.pone.0014211)

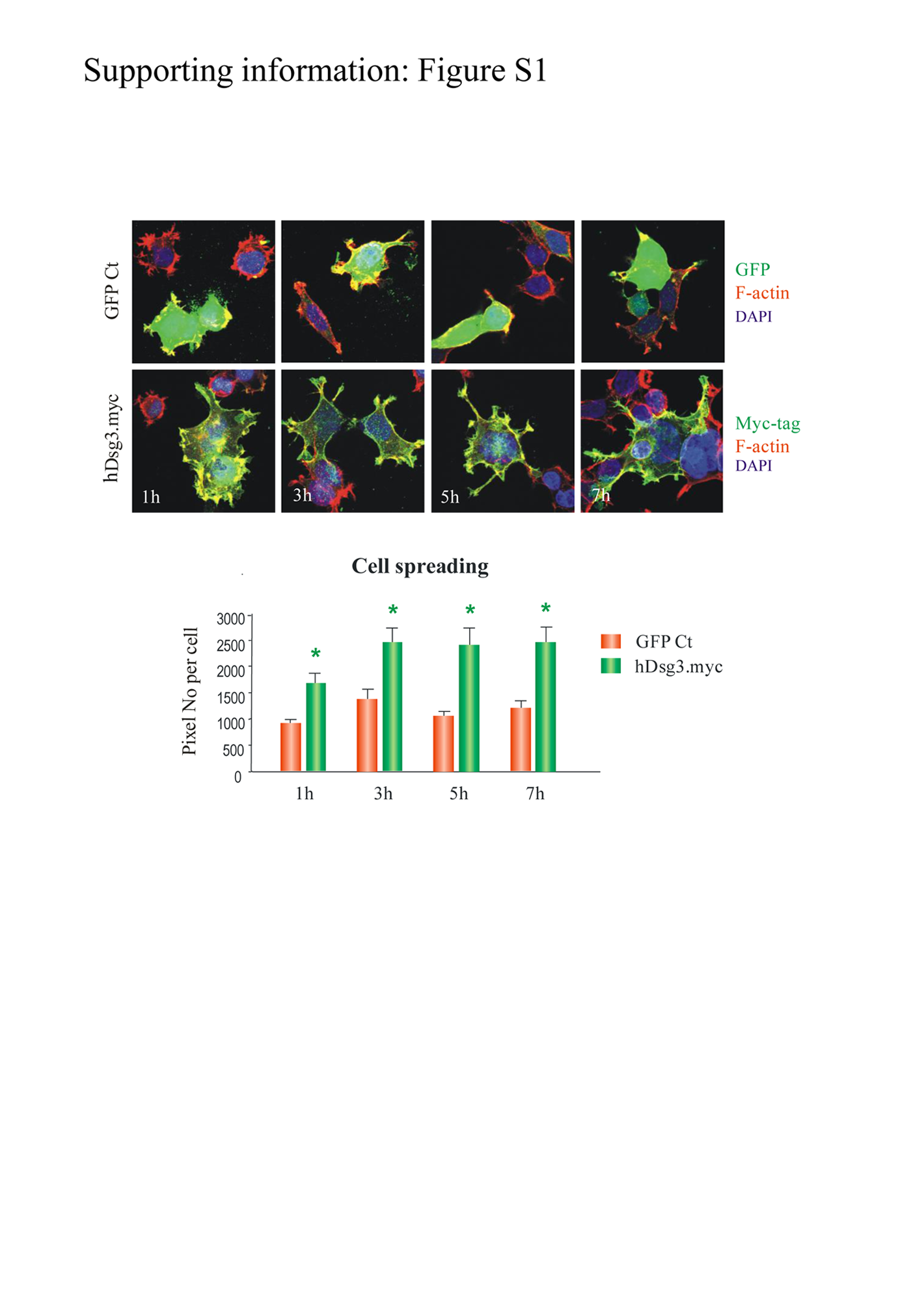

Supplement: Figure S1 — Expression of Dsg3 caused faster and wider spreading of 293T cells. Cells were transfected transiently with either the pBABE-GFP or -hDsg3.myc construct for two days before harvesting with trypsin. Cells then were seeded at low density onto coverslips without coating and fixed at various time points prior to immunostaining for the indicated proteins. Images were analysed in ImageJ software and an area of over 20 positively transfected cells in each group (pixel no per cell), from 4 arbitrary fields, was scored and presented as mean±sd (*p<0.002). (9.72 MB TIF) [file pone.0014211.s001.tif]

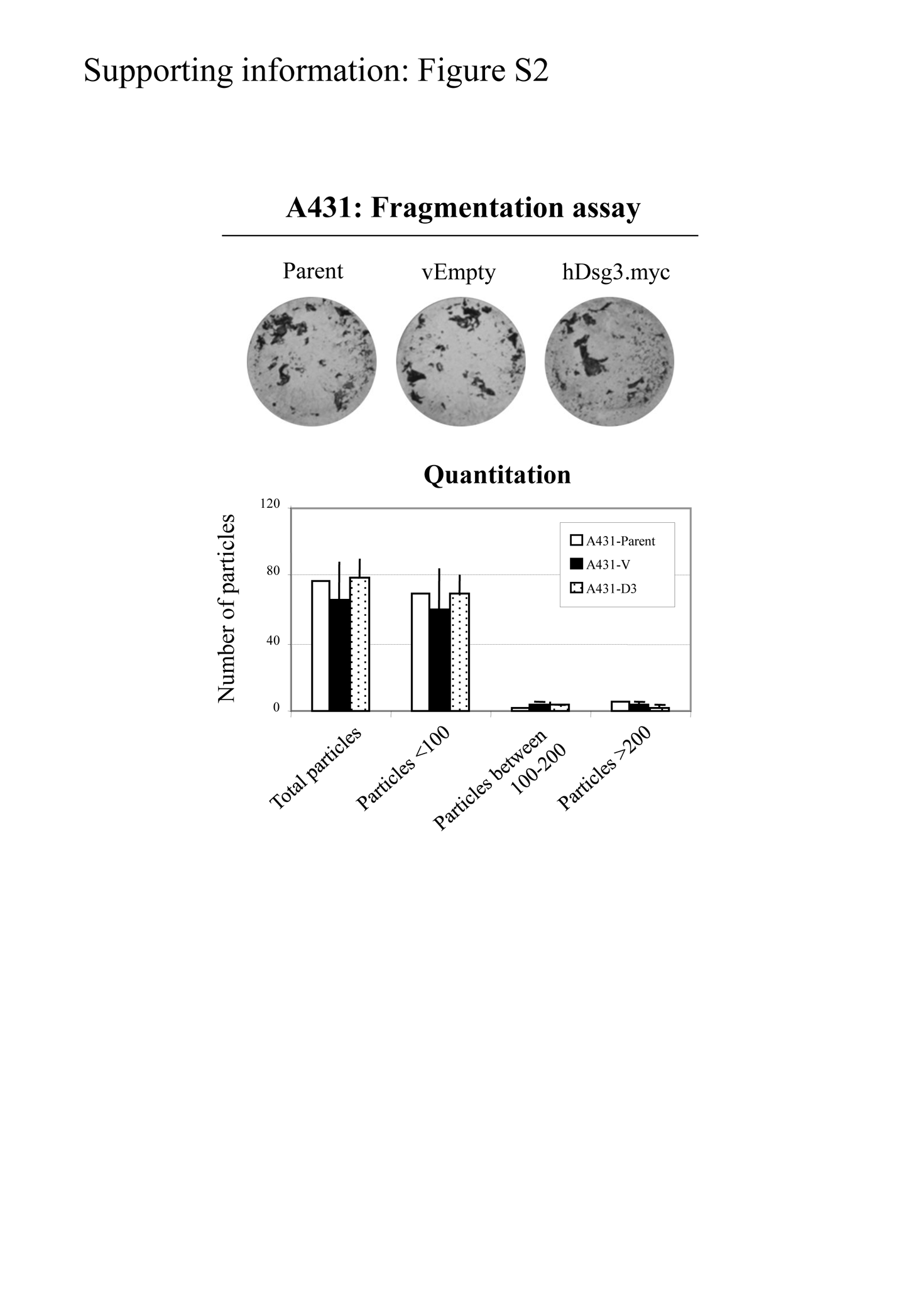

Supplement: Figure S2 — Over-expression of Dsg3 failed to enhance cell-cell adhesion. Cells were grown to confluence before being treated with 2.4 units/ml dispase for 20 minutes to detach the epithelial sheets. The epithelial sheets were washed with PBS, twice gently, before being subjected to mechanical stress by pipetting three-five times with 1-ml blue tips. The epithelial fragments were quantitated by ImageJ and presented as the mean+sd (Exp n = 6). The presented data were pooled from three independent experiments. (9.91 MB TIF) [file pone.0014211.s002.tif]

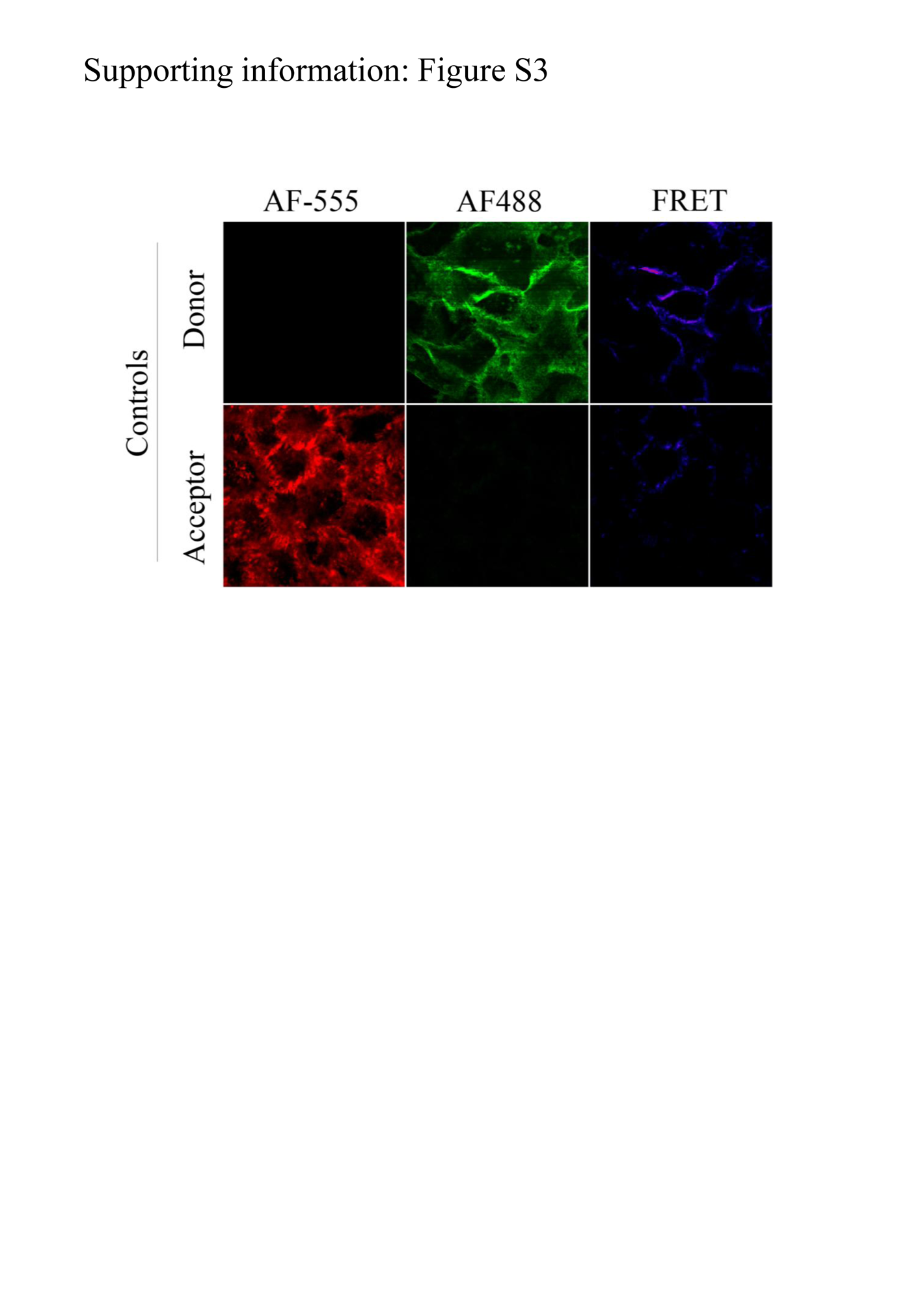

Supplement: Figure S3 — Control images for the AbFRET experiments, showing the level of bleed through into the FRET channel of cells labelled with the Donor alone and Acceptor alone. The FRET channel is shown using the Spectrum look up table from Image J. (9.53 MB TIF) [file pone.0014211.s003.tif]
